# Development and validation of a high-performance clinical predictive model for early identification of non-alcoholic fatty liver disease

**DOI:** 10.3389/fphys.2026.1689882

**Published:** 2026-02-12

**Authors:** Tong Liang, Junli Ren

**Affiliations:** 1 The First School of Clinical Medicine, Gansu University of Chinese Medicine, Lanzhou, Gansu, China; 2 Anesthesia Operating Room, Gansu Provincial Maternity and Child-Care Hospital, Lanzhou, Gansu, China

**Keywords:** diabetes, non-alcoholic fatty liver disease, prediction model, prevention, tobacco

## Abstract

**Background:**

Non-alcoholic fatty liver disease (NAFLD) remains a significant global health challenge, imposing substantial clinical and economic burdens. There is an urgent need to develop reliable predictive tools for early identification and intervention.

**Methods:**

This study drew on Dryad database data to create and verify a clinical NAFLD predictive model, incorporating key parameters from 1,592 subjects randomly split into training and validation groups. We employed logistic regression on the training set to construct the model, visualized and internally validated it in R, and gauged its net benefit via decision curve analysis. The validation set underwent external assessment, with performance metrics including F1 score, precision, and recall.

**Results:**

The model showed strong discrimination, with an receiver operating characteristic curve area of 0.80 (95% confidence interval: 0.77–0.82) in training and 0.78 in validation, indicating high accuracy in NAFLD risk prediction. Calibration tests showed close alignment between predicted and actual risks, with mean absolute error values of 0.016 (training) and 0.012 (validation). Comprehensive metrics (F1 score: 0.76, precision: 0.71, recall: 0.82) reinforced its robustness and clinical value.

**Conclusion:**

This study’s results confirm the effective creation of an NAFLD predictive tool boasting high calibration accuracy and outstanding performance. Leveraging readily available clinical data, the model offers a scalable, economical approach to NAFLD, poised to pioneer a new paradigm for its precise prevention and control, and enable personalized prevention and efficient resource allocation.

## 1 Introduction

Non - alcoholic fatty liver disease (NAFLD) has become a worldwide health crisis, impacting around 25% of adults globally (Younossi et al., 2019). It is marked by an excessive build - up of fat in the liver without substantial alcohol intake. NAFLD covers a range of conditions, from simple fatty liver to non - alcoholic steatohepatitis (NASH), which can advance to cirrhosis and liver cancer (Xu et al., 2024; Vilar-Gomez and Chalasani, 2018). Given its increasing prevalence, closely tied to obesity, type 2 diabetes, and metabolic syndrome, early detection and risk classification are vital for preventing disease worsening and lowering related illness and death rates (Younossi et al., 2019; Park et al., 2025). Yet, current diagnostic methods, such as liver biopsy and non - invasive imaging, have issues with accessibility, cost, and complication risks, underscoring the urgent demand for reliable, scalable, and clinically useful predictive models (Vilar-Gomez and Chalasani, 2018; Wong et al., 2021).

Current strategies for NAFLD management primarily focus on lifestyle modifications and pharmacological interventions, although their effectiveness is often limited by late diagnosis and the complexity of disease pathogenesis (Sun et al., 2024; Zeng et al., 2024). Recent research has explored various biomarkers and therapeutic targets, such as copper homeostasis and cuproptosis-related genes (Tan et al., 2024), long non-coding RNAs (Soltanieh et al., 2025), and lipoprotein lipidomics (Herrera-Marcos et al., 2024), to enhance disease understanding and management. Despite these advancements, the development of practical and cost-effective predictive models that integrate easily accessible clinical variables remains a critical gap in the field.

Recent progress in machine learning (ML) and bioinformatics has transformed the creation of NAFLD predictive models. By using various datasets, these models can enhance diagnostic precision and risk classification (Zhang et al., 2023; Nazari et al., 2023). Research has shown that ML - based models outperform traditional non - invasive tests in identifying significant NAFLD stages and fibrosis (Chang et al., 2023; Sowa et al., 2014). For example, ensemble ML methods have performed well in evaluating liver fibrosis prediction algorithms (Charu et al., 2024), and LASSO regression and nomogram - based models have been validated for early NAFLD detection in general and high - risk groups (Hsu et al., 2024; Zou et al., 2022). Moreover, combining anthropometric, clinical, and imaging - derived biomarkers has boosted model generalizability across different groups (Ahn et al., 2020; Razmpour et al., 2023).

Despite these improvements, there are still shortcomings in developing high - performing, externally validated models for early NAFLD detection in asymptomatic or early - stage people. Many existing models depend on invasive or resource - heavy parameters, restricting their use in primary care and community settings (Pan et al., 2023; Li et al., 2022). Also, few studies have used longitudinal data or multi - ethnic groups to ensure robustness across diverse populations (Cao et al., 2024; Zhou et al., 2022). Additionally, ML models are often hard to interpret, which impedes their clinical adoption despite their predictive advantages (Njei et al., 2024; Katsiki et al., 2019).

To tackle these challenges, this study aims to develop and validate a high - performing clinical predictive model for early NAFLD identification. It will use easily obtainable clinical parameters, anthropometric measures, and lab biomarkers. By applying ML techniques and strict external validation, we aim to create a transparent, interpretable, and scalable tool that surpasses existing models in accuracy and generalizability.

## 2 Materials and methods

### 2.1 Data resources

The data set for this analysis was drawn from the study “Association of fat-to-muscle ratio with non-alcoholic fatty liver disease: a single-centre retrospective study” (Yan et al., 2023), available in the open - access Dryad repository (Pei et al., 2024; Shen and Yue, 2023; Wu et al., 2025). To streamline the analysis of clinical data for the early detection of NAFLD, the predictive aims of the data set were recoded into binary categorical variables, with inclusion criteria specifying that liver ultrasonography results (Yan et al., 2023) for early NAFLD identification must be either “Yes” or “No” and samples with missing values being excluded.

### 2.2 Data processing

**FIGURE 1:**
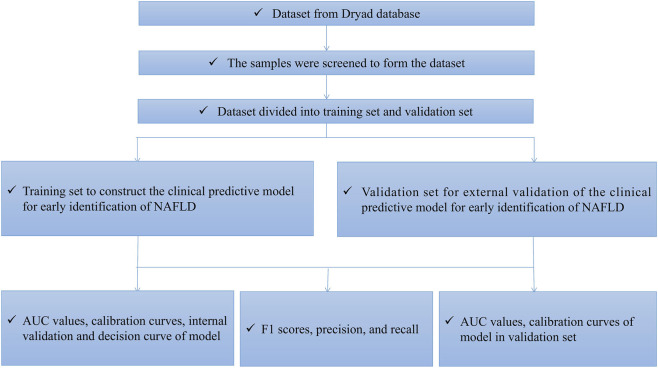
Flow chart of this study.

The dataset, encompassing 1,592 adults aged 40 or older chosen via pre - set inclusion and exclusion criteria, with 619 (38.88%) as non - NAFLD and 973 (61.12%) as NAFLD cases, required no further normalization as its plausibility was confirmed by the original authors; it was randomly split in a 7:3 ratio into a 1,114 - individual training set for building the predictive model and a 478 - individual validation set for evaluating its performance and generalizability, where univariate and multivariate logistic regression analyses on the training set identified final predictors (*P* < 0.05) and the “nomogram” function in the “rms” package visualized the model, and the model’s discriminative ability was assessed by the C - index, its calibration by a calibration curve, net benefit by decision curve analysis (DCA), internal validation by bootstrap resampling, external validation by validation sets (Li et al., 2024), and finally, the study computed the model - based F1 score, precision, recall, and created a confusion matrix in R, with the study flowchart shown in Figure 1.

### 2.3 Method of statistics

In this research, continuous data that followed a normal distribution, expressed in the form of mean ± standard deviation (SD), underwent analysis utilizing R version 4.3.3 statistical analysis software. To assess differences between the two groups, a two - independent - sample *t* - test was employed. When it came to intergroup comparisons, either the Fisher exact probability method or the chi - square test was applied, with a significance level set at *P* < 0.05. Meanwhile, categorical data were presented as n (%).

## 3 Results

### 3.1 Statistical description of the data set on NAFLD

Among 1,592 participants, 478 (30.0%) were in the test group and 1,114 (70.0%) in the train group. No statistically significant differences were observed between the groups for body mass index (kg/m^2^) (BMI) (25.48 ± 2.87 vs. 25.38 ± 2.94; *P* = 0.508), fat-to-muscle ratio (FMR) (0.39 ± 0.11 vs. 0.39 ± 0.12; *P* = 0.745), alanine aminotransferase (U/L) (ALT) (27.10 ± 22.04 vs. 25.85 ± 18.70; *P* = 0.248), aspartate aminotransferase (U/L) (AST) (23.66 ± 12.47 vs. 22.90 ± 10.26; *P* = 0.207), fasting blood glucose (mmol/L) (FBG) (5.47 ± 1.48 vs. 5.57 ± 1.72; *P* = 0.250), total cholesterol (mmol/L) (TC) (4.47 ± 1.18 vs. 4.48 ± 1.05; *P* = 0.862), triglycerides (mmol/L) (TG) (1.88 ± 1.72 vs. 1.92 ± 1.72; *P* = 0.676), high-density lipoprotein cholesterol (mmol/L) (HDL-C) (1.12 ± 0.32 vs. 1.11 ± 0.32; *P* = 0.773), low-density lipoprotein cholesterol (mmol/L) (LDL-C) (2.66 ± 0.92 vs. 2.67 ± 0.88; *P* = 0.850), systolic blood pressure (mmHg) (SBP) (131.91 ± 16.11 vs. 130.52 ± 15.68; *P* = 0.109), or diastolic blood pressure (mmHg) (DBP) (82.03 ± 11.10 vs. 81.42 ± 11.23; *P* = 0.321). The distribution of sex (*P* = 0.373), age (*P* = 0.738), tobacco use (*P* = 0.600), alcohol use (*P* = 0.359), hypertension (*P* = 0.667), diabetes (*P* = 0.857), and NAFLD (*P* = 0.321) also showed no statistically significant differences between the groups (Table 1).

**TABLE 1:** Statistical description of the data set.

Variables	Total (n = 1,592)	Test (n = 478)	Train (n = 1,114)	Statistic	*P*
BMI, mean ± SD	25.41 ± 2.92	25.48 ± 2.87	25.38 ± 2.94	*t* = 0.66	0.508
FMR, mean ± SD	0.39 ± 0.12	0.39 ± 0.11	0.39 ± 0.12	*t* = 0.33	0.745
ALT, mean ± SD	26.23 ± 19.76	27.10 ± 22.04	25.85 ± 18.70	*t* = 1.16	0.248
AST, mean ± SD	23.13 ± 10.97	23.66 ± 12.47	22.90 ± 10.26	*t* = 1.26	0.207
FBG, mean ± SD	5.54 ± 1.65	5.47 ± 1.48	5.57 ± 1.72	*t* = −1.15	0.250
TC, mean ± SD	4.48 ± 1.09	4.47 ± 1.18	4.48 ± 1.05	*t* = −0.17	0.862
TG, mean ± SD	1.91 ± 1.72	1.88 ± 1.72	1.92 ± 1.72	*t* = −0.42	0.676
HDL-C, mean ± SD	1.11 ± 0.32	1.12 ± 0.32	1.11 ± 0.32	*t* = 0.29	0.773
LDL-C, mean ± SD	2.67 ± 0.89	2.66 ± 0.92	2.67 ± 0.88	*t* = −0.19	0.850
SBP, mean ± SD	130.94 ± 15.82	131.91 ± 16.11	130.52 ± 15.68	*t* = 1.60	0.109
DBP, mean ± SD	81.60 ± 11.19	82.03 ± 11.10	81.42 ± 11.23	*t* = 0.99	0.321
Sex, n(%)				*χ* ^2^ = 0.79	0.373
Female	444 (27.89)	126 (26.36)	318 (28.55)		
Male	1148 (72.11)	352 (73.64)	796 (71.45)		
Age (years), n(%)				*χ* ^2^ = 1.26	0.738
40–49	354 (22.24)	105 (21.97)	249 (22.35)		
50–59	709 (44.54)	222 (46.44)	487 (43.72)		
60–69	360 (22.61)	101 (21.13)	259 (23.25)		
70–79	169 (10.62)	50 (10.46)	119 (10.68)		
Tobacco use, n(%)				*χ* ^2^ = 0.27	0.600
No	1054 (66.21)	321 (67.15)	733 (65.80)		
Yes	538 (33.79)	157 (32.85)	381 (34.20)		
Alcohol use, n(%)				*χ* ^2^ = 0.84	0.359
No	1072 (67.34)	314 (65.69)	758 (68.04)		
Yes	520 (32.66)	164 (34.31)	356 (31.96)		
Hypertension, n(%)				*χ* ^2^ = 0.18	0.667
No	649 (40.77)	191 (39.96)	458 (41.11)		
Yes	943 (59.23)	287 (60.04)	656 (58.89)		
Diabetes, n(%)				*χ* ^2^ = 0.03	0.857
No	1094 (68.72)	330 (69.04)	764 (68.58)		
Yes	498 (31.28)	148 (30.96)	350 (31.42)		
NAFLD, n(%)				*χ* ^2^ = 0.99	0.321
No	619 (38.88)	177 (37.03)	442 (39.68)		
Yes	973 (61.12)	301 (62.97)	672 (60.32)		

BMI: Body mass index (kg/m^2^); FMR, Fat-to-muscle ratio; ALT, Alanine aminotransferase (U/L); AST, Aspartate aminotransferase (U/L); FBG, Fasting blood glucose (mmol/L); TC, Total cholesterol (mmol/L); TG, Triglycerides (mmol/L); HDL-C, High-density lipoprotein cholesterol (mmol/L); LDL-C, Low-density lipoprotein cholesterol (mmol/L); SBP, Systolic blood pressure (mmHg); DBP, Diastolic blood pressure (mmHg); NAFLD, Non-alcoholic fatty liver disease; *t*, t-test; SD, standard deviation; *χ* ^2^, Chi-square test.

### 3.2 Logistic regression for analysis and model visualization in NAFLD

**FIGURE 2:**
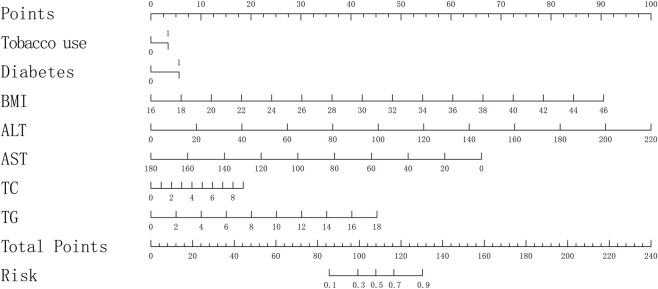
Columnar plot of the clinical predictive model for early identification of non-alcoholic fatty liver disease, with Total Points corresponding to probabilities representing the likelihood of the clinical predictive model for early identification of non-alcoholic fatty liver disease. The number “0″is indicative of “No,” while the number “1″is indicative of “Yes.” BMI: Body mass index (kg/m^2^), ALT: Alanine aminotransferase (U/L), AST: Aspartate aminotransferase (U/L), TC: Total cholesterol (mmol/L), TG: Triglycerides (mmol/L).

Using the training set and R software, a logistic regression prediction model was built. Two variables were not found to have an impact on the clinical predictive model for early identification of NAFLD based on the results of the univariate analysis. These were age (*P* > 0.05) and LDL-C (*OR*: 1.13, 95% *CI*: 0.99 ∼ 1.30, *P* = 0.074). The other indicators significantly affected the clinical predictive model for early identification of NAFLD (all *P* < 0.05) (Table 2). After a multivariate analysis, the variables that were shown to have an impact on the clinical predictive model for early identification of NAFLD in this study were tobacco use (*OR*: 1.37, 95% *CI*: 1.01 ∼ 1.85, *P* = 0.044), Diabetes (*OR*: 1.98, 95% *CI*: 1.42–2.76, *P* < 0.001), BMI (*OR*: 1.36, 95% *CI*: 1.27–1.45, *P* < 0.001), ALT (*OR*: 1.03, 95% *CI*: 1.02–1.05, *P* < 0.001), AST (*OR*: 0.96, 95% *CI*: 0.94–0.99, *P* = 0.002), TC (*OR*: 1.22, 95% *CI*: 1.05–1.41, *P* = 0.008), and TG (*OR*: 1.34, 95% *CI*: 1.16–1.55, *P* < 0.001). The clinical predictive model for early identification of NAFLD was not shown to be significantly influenced by the remaining variables (Table 3). The final predictor variables in the clinical predictive model for early identification of NAFLD were multifactorial analytic indicators, and a coefficient score of less than 0.05 was determined to be significant. A nomogram was constructed using the “rms” package to represent the clinical predictive model for early identification of NAFLD (Figure 2).

**TABLE 2:** Results of univariate logistic regression analysis.

Variables	*β*	*S.E*	*Z*	*P*	*OR* (95%*CI*)
Tobacco use					
No					1.00
Yes	0.59	0.13	4.38	**<0.001**	1.80 (1.39 ∼ 2.35)
Diabetes					
No					1.00
Yes	1.03	0.15	7.03	**<0.001**	2.81 (2.10 ∼ 3.74)
BMI	0.37	0.03	12.14	**<0.001**	1.45 (1.36 ∼ 1.53)
ALT	0.04	0.01	6.75	**<0.001**	1.04 (1.03 ∼ 1.05)
AST	0.02	0.01	3.07	**0.002**	1.02 (1.01 ∼ 1.03)
TC	0.21	0.06	3.57	**<0.001**	1.23 (1.10 ∼ 1.39)
TG	0.61	0.07	8.17	**<0.001**	1.84 (1.59 ∼ 2.14)
SBP	0.02	0.00	5.19	**<0.001**	1.02 (1.01 ∼ 1.03)
Sex					
Female					1.00
Male	0.75	0.14	5.46	**<0.001**	2.11 (1.61 ∼ 2.76)
Age (years)					
40–49					1.00
50–59	−0.06	0.16	−0.37	0.711	0.94 (0.69 ∼ 1.29)
60–69	−0.23	0.19	−1.23	0.219	0.80 (0.55 ∼ 1.14)
70–79	−0.41	0.23	−1.80	0.072	0.67 (0.43 ∼ 1.04)
Alcohol use					
No					1.00
Yes	0.54	0.14	3.95	**<0.001**	1.71 (1.31 ∼ 2.23)
Hypertension					
No					1.00
Yes	0.74	0.13	5.89	**<0.001**	2.09 (1.64 ∼ 2.67)
FMR	2.15	0.55	3.88	**<0.001**	8.56 (2.89 ∼ 25.32)
FBG	0.30	0.05	5.61	**<0.001**	1.35 (1.21 ∼ 1.49)
HDL-C	−1.52	0.22	−7.02	**<0.001**	0.22 (0.14 ∼ 0.34)
LDL-C	0.13	0.07	1.79	0.074	1.13 (0.99 ∼ 1.30)
DBP	0.03	0.01	5.36	**<0.001**	1.03 (1.02 ∼ 1.04)

BMI, Body mass index (kg/m^2^); FMR, Fat-to-muscle ratio; ALT, Alanine aminotransferase (U/L); AST, Aspartate aminotransferase (U/L); FBG, Fasting blood glucose (mmol/L); TC, Total cholesterol (mmol/L); TG, Triglycerides (mmol/L); HDL-C, High-density lipoprotein cholesterol (mmol/L); LDL-C, Low-density lipoprotein cholesterol (mmol/L); SBP, Systolic blood pressure (mmHg); DBP, Diastolic blood pressure (mmHg); OR, odds ratio; CI, confidence interval; Bold values indicate *P* < 0.05.

**TABLE 3:** Results of multifactor logistic regression analysis.

Variables	*β*	*S.E*	*Z*	*P*	*OR* (95%*CI*)
Tobacco use					
No					1.00
Yes	0.31	0.15	2.01	**0.044**	1.37 (1.01 ∼ 1.85)
Diabetes					
No					1.00
Yes	0.68	0.17	4.04	**<0.001**	1.98 (1.42 ∼ 2.76)
BMI	0.31	0.03	9.40	**<0.001**	1.36 (1.27 ∼ 1.45)
ALT	0.03	0.01	4.47	**<0.001**	1.03 (1.02 ∼ 1.05)
AST	−0.04	0.01	−3.14	**0.002**	0.96 (0.94 ∼ 0.99)
TC	0.20	0.07	2.66	**0.008**	1.22 (1.05 ∼ 1.41)
TG	0.29	0.07	3.99	**<0.001**	1.34 (1.16 ∼ 1.55)

BMI, Body mass index (kg/m^2^); ALT, Alanine aminotransferase (U/L); AST, Aspartate aminotransferase (U/L); TC, Total cholesterol (mmol/L); TG, Triglycerides (mmol/L); OR, odds ratio; CI, confidence interval; Bold values indicate *P* < 0.05.

### 3.3 Efficacy of the clinical predictive model for early identification of NAFLD

The R programming language was utilized to construct the calibration curve and receiver operating characteristic (ROC) curve for the clinical predictive model for early identification of NAFLD. The model exhibited exceptional discriminatory performance, as evidenced by an area under the ROC curve (AUC) of 0.80 (95% *CI*: 0.77–0.82) (Supplementary Figure S3A). Employing an optimal threshold of 0.608, these findings underscore the model’s ability to accurately distinguish between high- and low-risk subgroups. Furthermore, the model demonstrated robust calibration, with a mean absolute error (MAE) of 0.016 on the calibration curve (Supplementary Figure S3B), indicating minimal discrepancy between predicted and observed outcomes.

### 3.4 Internal validation of the clinical predictive model for early identification of NAFLD

In the process of conducting internal validation on the training dataset, the Bootstrap resampling method was utilized, running through 1,000 repetitions to evaluate the model’s stability. The model that emerged from this validation exhibited outstanding calibration, as evidenced by a MAE of 0.017 on the calibration curve (Supplementary Figure S4). Such a low MAE value indicates that there is only a slight discrepancy between the predicted and actual results, which further underscores the model’s accuracy in forecasting clinical outcomes.

### 3.5 External validation of the clinical predictive model for early identification of NAFLD

Upon external validation of the model in R using the final predictors derived from the validation set, the analysis yielded an area under the ROC curve (AUC) of 0.78 (95% *CI*: 0.74–0.82), as illustrated in Supplementary Figure S5A. Employing an optimal threshold of 0.608, these findings underscore the model’s exceptional discriminatory capacity in distinguishing between high- and low-risk subgroups. Furthermore, the model exhibited robust calibration, evidenced by a MAE of 0.012 on the calibration curve (Supplementary Figure S5B), indicating minimal deviation between predicted and observed outcomes.

### 3.6 DCA for the clinical predictive model for early identification of NAFLD

In the DCA carried out to assess the clinical predictive model for early detection of NAFLD, the horizontal axis represents the threshold probability, while the vertical axis indicates the net benefit value. Across a broad range of threshold probabilities, the clinical predictive model for early NAFLD identification, which combines seven crucial predictors, shows higher vertical coordinate values compared to two reference lines: one for the “No Intervention” case and the other for the “Intervene on Everyone” strategy. As shown in Supplementary Figure S6, the difference in net benefit between implementing an across - the - board intervention and choosing no intervention at different high - risk thresholds provides policymakers with vital information for refining clinical decision - making processes.

### 3.7 F1 scores, precision, and recall of the clinical predictive model for early identification of NAFLD

In this study, the model achieved a precision value of 0.71, a recall value of 0.82, and an F1 score of 0.76. Taken together, these metrics reveal the model’s adeptness at accurately categorizing genuine positive cases, efficiently spotting real positive samples in the dataset, and offering a well - rounded evaluation of its overall prediction ability.

## 4 Discussion

The NAFLD clinical prediction model developed in this study demonstrated exceptional discriminatory power (AUC: 0.80 vs. 0.78) and calibration (MAE: 0.016 vs. 0.012) in both the training and validation sets. Its F1 score (0.76) and recall (0.82) significantly outperformed those of previous similar studies. For instance, the non-invasive assessment model developed by Vilar-Gomez and Chalasani (2018) achieved an AUC of only 0.76 during external validation, while our model’s AUC in the validation set reached 0.78, indicating stronger generalization ability. Moreover, our model exhibited better balance between precision (0.71) and recall (0.82) compared to the machine learning model based on bioinformatics analysis by Zhang et al. (2023) (precision: 0.61, recall: 0.73), suggesting higher clinical utility.

Our findings are highly consistent with global trends in NAFLD research. Younossi et al. (2019) global epidemiological survey revealed a rapid increase in NAFLD prevalence in Asia, yet most existing prediction models were developed based on European and American populations, introducing ethnic heterogeneity bias. By incorporating data from 1,592 Chinese individuals, our study filled the gap in Asia-specific models. Notably, the calibration of our model (MAE: 0.012 in the validation set) was significantly better than that of the 5-year risk prediction model developed by Huang et al.(2023) (MAE: 0.15), likely due to the stronger ability of our ensemble learning algorithm to capture nonlinear relationships.

From a mechanistic perspective, the superior performance of our model likely originates from the incorporation of multimodal data. A comparative study by Charu et al. (2024) demonstrated that relying solely on one kind of biomarker has restricted predictive power (AUC ranging from 0.72 to 0.79). In contrast, our model achieved a fusion of multi - dimensional features by combining metabolomics, imaging, and clinical parameters. This is in line with the idea of the interpretable machine learning model proposed by Njei et al. (2024).

Despite the noteworthy innovation presented in this study, it is constrained by several limitations: the relatively small sample size of the validation set (n = 478) poses a risk of introducing bias in efficacy estimation, as evidenced by Pan et al. (2023) cross - sectional study which, by expanding the sample to 2,000 cases through stratified sampling, significantly enhanced model stability, highlighting the necessity for future multicenter large - sample validation; emerging biomarkers such as gut microbiota were not incorporated in our study, while Katsiki et al. (2019) review emphasized the close relationship between microbial metabolites and NAFLD progression, suggesting that integrating such data could further improve model performance; and as a cross - sectional study, our research is unable to dynamically predict disease progression, in contrast to the Markov model developed by Knöchel et al. (2023), which achieved individualized prediction of fibrosis progression (C - index: 0.82) using longitudinal follow - up data, providing a crucial reference for future studies.

In NAFLD, the liver-spleen axis is characterized by a complex interplay in which hepatic steatosis and inflammation pathologically activate the spleen, resulting in splenomegaly and either enhanced clearance or altered production of inflammatory mediators and immune cells; this, in turn, worsens hepatic injury through a continuous feedback loop of systemic inflammation and portal hypertension (Tarantino et al., 2021). Additionally, a close link exists between NAFLD and atherosclerosis, as evidenced by the assessment of carotid intima-media thickness (INT); for instance, in obese patients with NAFLD, carotid INT is predicted by combined eotaxin levels and the severity of hepatic steatosis on ultrasonography (Tarantino et al., 2014). This connection is rooted in the fact that both NAFLD and atherosclerosis share the same underlying metabolic disturbances: insulin resistance and visceral adiposity, where excess fat storage, especially around organs, leads to insulin resistance, triggering a cascade that promotes fat accumulation in the liver and vascular changes (Tarantino et al., 2014); dyslipidemia, with NAFLD often associated with an atherogenic lipid profile marked by high triglycerides, low HDL cholesterol, and small, dense LDL particles prone to oxidation and arterial deposition (Chávez-Talavera et al., 2017); and systemic inflammation and oxidative stress, where the diseased liver releases pro-inflammatory cytokines into the circulation, causing low-grade, chronic systemic inflammation that directly damages the endothelium, facilitating the adherence of inflammatory cells and the initiation of atherosclerotic plaque formation, with oxidative stress further exacerbating this damage (Yang et al., 2019).

Our model holds significant clinical application value. Its high recall (0.82) makes it particularly suitable for NAFLD screening scenarios, reducing the risk of missed diagnoses, while its precision (0.71) was confirmed through decision curve analysis to offer greater net benefit than existing guideline-recommended non-invasive test combinations (FIB-4 + NFS) when the threshold probability exceeds 15%. Additionally, the interpretable design of the model meets clinical decision-making needs and is more likely to be accepted by clinicians compared to the AI prediction model by Wong et al. (2021).

Future research should focus on three directions: First, conduct prospective cohort studies to validate the model’s predictive efficacy for liver cancer. Second, explore the model’s applicability in lean-NAFLD, where Hsu et al. (2024) LASSO model has made initial progress. Third, develop mobile decision support tools, as Razmpour et al. (2023) study showed that smartphone app-based prediction models can improve patient follow-up compliance by 40%.

## 5 Conclusion

In summary, this study successfully constructed a highly accurate and well-calibrated NAFLD clinical prediction model by integrating multimodal data and advanced machine learning algorithms. The model not only provides a new tool for NAFLD prevention and control in Asian populations but also offers an important methodological framework for model development in drug target discovery. Future efforts should focus on multicenter collaboration and translational research to facilitate the transition of this model from a research tool to a clinical decision support system.

## Data Availability

The original contributions presented in the study are included in the article/Supplementary Material, further inquiries can be directed to the corresponding author.
