# Supplementary figures and images for: Development and validation of a high-performance clinical predictive model for early identification of non-alcoholic fatty liver disease
Source: Front Physiol. 2026 Feb 12;17:1689882. doi: 10.3389/fphys.2026.1689882 (PMC12935685; doi:10.3389/fphys.2026.1689882)

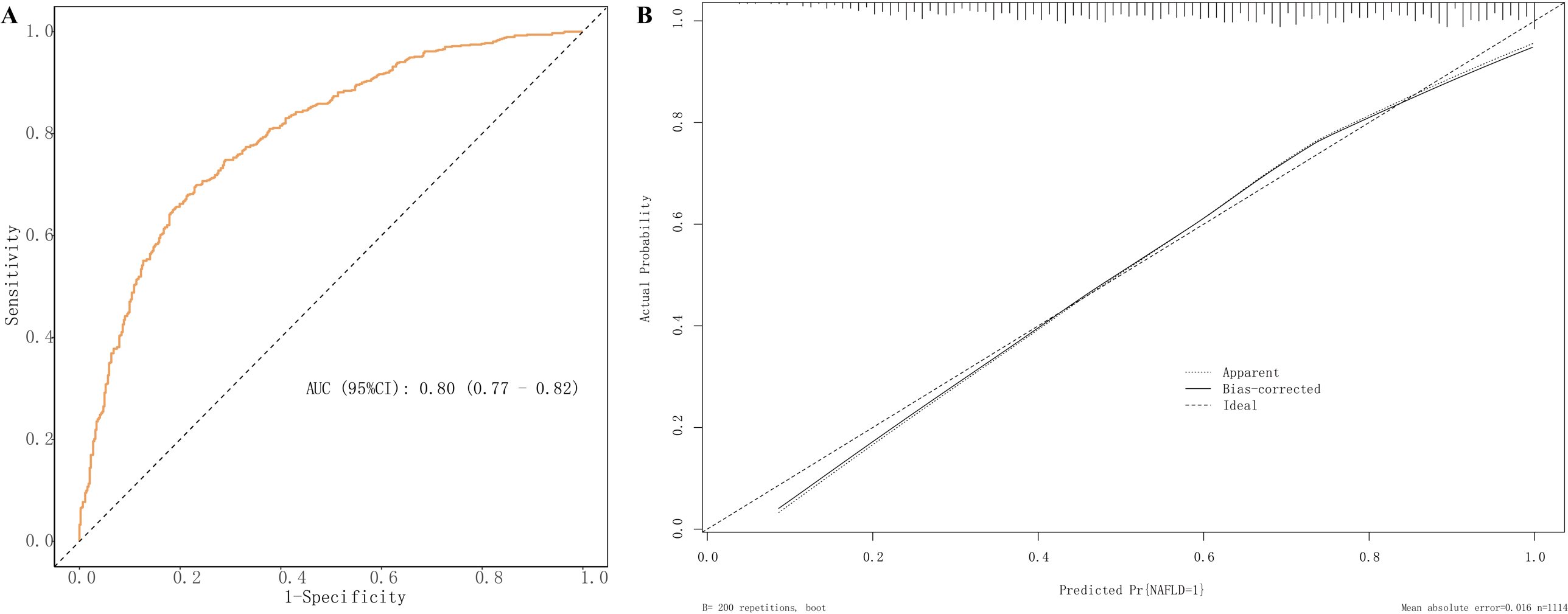

Supplement: Supplementary file 2 [file Image3.tif]

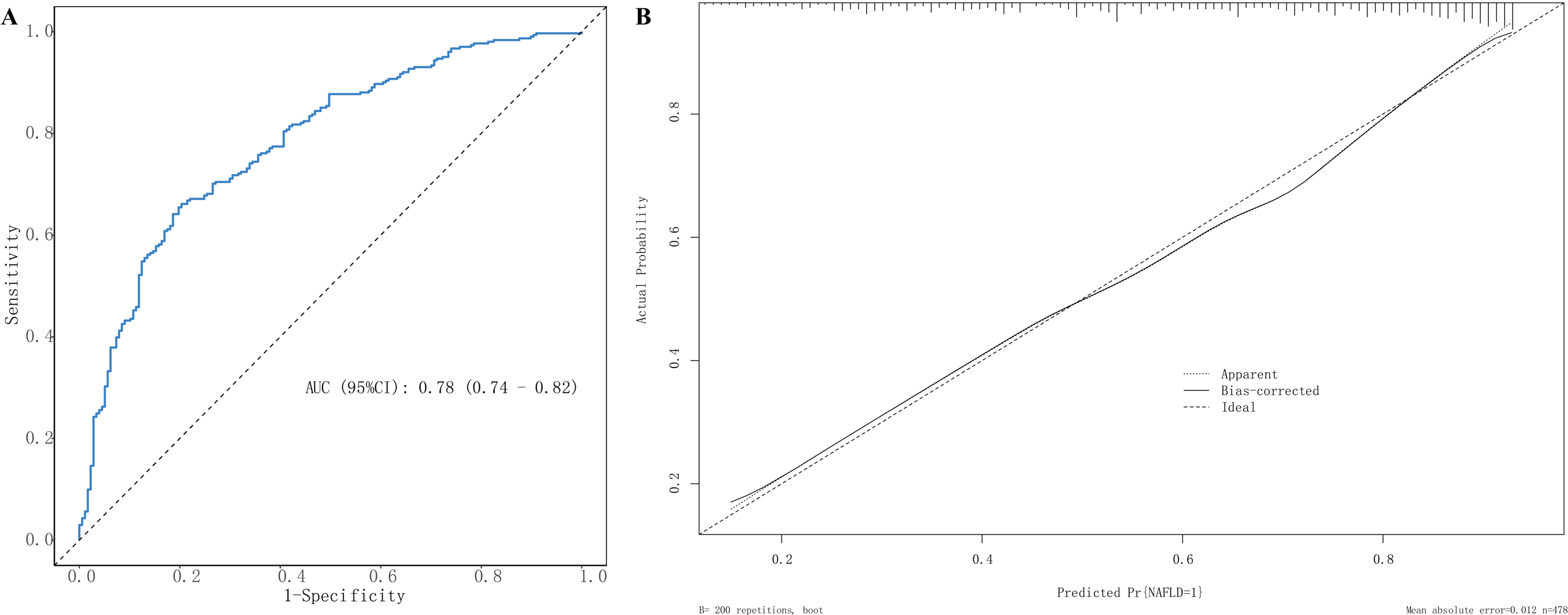

Supplement: Supplementary file 3 [file Image4.tif]
